# Optimisation of analytical methods for tuberculosis drug detection in wastewater: A multinational study

**DOI:** 10.1016/j.heliyon.2024.e30720

**Published:** 2024-05-07

**Authors:** Hlengiwe N. Mtetwa, Isaac D. Amoah, Sheena Kumari, Faizal Bux, Poovendhree Reddy

**Affiliations:** a Department of Community Health Studies, Faculty of Health Sciences, Durban University of Technology, PO Box 1334, Durban, 4000, South Africa; b Institute for Water and Wastewater Technology (IWWT), Durban University of Technology, PO Box 1334, Durban, 4000, South Africa; c The University of Arizona, The Department of Environmental Science, Shantz Building Rm 4291177 E 4th St, Tucson, AZ, 85721, USA

**Keywords:** Tuberculosis drugs, wastewater analysis, Chromatography, Wastewater-based epidemiology

## Abstract

Wastewater-based epidemiology (WBE) is a robust tool for disease surveillance and monitoring of pharmaceutical consumption. However, monitoring tuberculosis (TB) drug consumption faces challenges due to limited data availability. This study aimed to optimise methods for detecting TB drugs in treated and untreated wastewater from four African countries: South Africa, Nigeria, Kenya, and Cameroon. The limit of detection (LOD) for these drugs ranged from a minimum of 2.20 (±1.02) for rifampicin to a maximum of 2.95 (±0.79) for pyrazinamide. A parallel trend was observed concerning the limit of quantification (LOQ), with rifampicin reporting the lowest average LOQ of 7.33 (±3.44) and pyrazinamide showing the highest average LOQ of 9.81 (±2.64). The variance in LOD and LOQ values could be attributed to factors such as drug polarity. Erythromycin and rifampicin exhibited moderately polar LogP values (2.6 and 2.95), indicating higher lipid affinity and lower water affinity.

Conversely, ethambutol, pyrazinamide, and isoniazid displayed polar LogP values (−0.059, −0.6, and −0.7), suggesting lower lipid affinity and greater water affinity. The study revealed that storing wastewater samples for up to 5 days did not result in significant drug concentration loss, with concentration reduction remaining below 1 log throughout the storage period. Application of the optimised method for drug detection and quantification in both treated and untreated wastewater unveiled varied results. Detection frequencies varied among drugs, with ethambutol consistently most detected, while pyrazinamide and isoniazid were least detected in wastewater from only two countries. Most untreated wastewater samples had undetectable drug concentrations, ranging from 1.21 ng/mL for erythromycin to 54.61 ng/mL for isoniazid. This variability may suggest differences in drug consumption within connected communities. In treated wastewater samples, detectable drug concentrations ranged from 1.27 ng/mL for isoniazid to 10.20 ng/mL for ethambutol. Wastewater treatment plants exhibited variable removal efficiencies for different drugs, emphasising the need for further optimisation. Detecting these drugs in treated wastewater suggests potential surface water contamination and subsequent risks of human exposure, underscoring continued research's importance.

## Highlights

• Methods were optimised to detect TB drugs in African wastewater samples.
• The TB drugs exhibited variation in polarity, ranging from moderate to high polarity.
• Storage of wastewater samples for up to 5 days had an insignificant impact on drug concentrations.
• The detection and concentration of drugs in samples from various countries varied.
• There were variable removal efficiencies in wastewater treatment plants for different TB drugs.

## 1 Introduction

Tuberculosis (TB), a disease caused by the bacterium *Mycobacterium tuberculosis*, continues to be a formidable global health crisis. Despite advancements in medicine and public health, it inflicts millions with infection and results in a significant number of deaths annually consumption [1]. The cornerstone of TB treatment has traditionally been a regimen of antibiotics, including isoniazid, rifampicin, ethambutol, and pyrazinamide. However, the efficacy of these treatments is increasingly jeopardised by the emergence and spread of antibiotic-resistant TB strains. The inadequacies of conventional monitoring systems further compound this burgeoning antibiotic resistance crisis. Such systems often fail to account for the entire affected population, mainly due to the prevalence of self-medication, the existence of informal drug markets, and inconsistent access to formal healthcare services ([2,3]; [4]). These gaps highlight the urgent need for innovative and comprehensive methods to monitor antibiotic use and resistance patterns.

In response to this challenge, current efforts have turned towards Wastewater-based Epidemiology (WBE) ([5,6]; [7]; [8]). WBE is a novel approach that analyses pharmaceutical residues in urban wastewater to gather real-time, non-invasive insights into community-wide drug consumption patterns [9,10,60]. This method has the potential to offer a more precise and inclusive understanding of antibiotic use, which is particularly beneficial in areas where healthcare access is sporadic or non-existent [[11], [12], [13]].

Building upon the foundation of WBE, this study endeavours to specifically tailor and enhance the technique for the surveillance of TB antibiotics. It focuses on refining the detection, analysis, and understanding of the presence and movement of TB antibiotics within wastewater systems [14]. The aim is to overcome existing limitations in the methodology, thereby increasing data reliability concerning drug consumption. Such improvements could significantly influence public health strategies, enabling the formulation of more effective interventions, the development of informed prescription guidelines, and the early identification of resistance trends.

The study seeks to highlight its concentrated application of WBE to the surveillance of TB antibiotics—a domain still in its nascent stages but with immense potential for impact. Through advancing methods for detecting and analysing TB antibiotics in wastewater, the study is poised to provide vital insights into drug consumption patterns. The ultimate goal is to bolster TB treatment outcomes, curb the spread of antibiotic resistance, and fortify the global fight against tuberculosis. This marks a significant stride towards more productive and adaptable tuberculosis control and prevention strategies at the population level.

## 2 Materials and methods

### 2.1 Study location

Wastewater samples were collected from four African countries: Cameroon, Nigeria, South Africa and Kenya. These countries were chosen to illustrate different regions in Sub-Saharan Africa, including West Africa (Nigeria), Central Africa (Cameroon), East Africa (Kenya), and Southern Africa (South Africa). In each country, one wastewater treatment plant (WWTP) was selected for sampling, except for South Africa, where three WWTPs were included due to their accessibility and availability of logistical support (Table 1). WWTPs were selected based on their capacity to serve a minimum of 5000 individuals and their treatment of hospital sewage, as detailed in (Mtetwa et al., 2023). These hospitals are designated centres for TB treatment. Therefore, selecting these WWTPs increased the probability of detection of the drugs, which is critical for the method optimisation.

**Table 1:** Details about the wastewater treatment facilities examined in this study.

WWTP	Design Capacity(Mℓ/d)	Remarks
WWTP A	18.8	Provides healthcare services to the community through affiliated clinics. Receives wastewater support from them.
WWTP B	4.90	Functions as both a facility that receives patients and one that refers them to hospitals and clinics.
WWTP C	70.0	Committed to delivering specialized care for challenging cases of MDR-TB.
WWTP D	0.801	Receives domestic sewage from social housing, not from hospitals, livestock farms, or the pharmaceutical industry.
WWTP E	7.95	Receives input from two hospitals but does not receive input from pharmaceutical industries or major animal farms.
WWTP F	0.817	Receives wastewater discharged by a teaching hospital.

The information presented in this table has been sourced from the studies conducted by Mtetwa et al. [15] and Mtetwa et al. [16].

### 2.2 Sampling and sample storage

Composite samples (1L) were collected from the influent (untreated wastewater) and final(post-chlorinated) effluent at each wastewater treatment plant (WWTP). Multiple subsamples of 100 mL each were taken at 30-s intervals to obtain the composite samples until the final volume of 1L was achieved. The samples were stored at four °C and shipped from the five African countries to the Institute for Water and Wastewater Technology (IWWT), Durban University of Technology in South Africa, using a commercial courier service. As soon as the samples were received, they were filtered through 0.45 m syringe filters and stored at −80 °C in 50 mL aliquots. The samples underwent additional filtration using 0.22 μm syringe filters before analysis using Liquid chromatography-mass spectrometry/mass spectrometry.

### 2.3 Chemicals and reagents

The solvents and reagents used for LC-MS/MS analysis were all of 99 % purity. Methanol water and formic acid were purchased from Sigma Aldrich. Standards for Erythromycin (ERY1) (molecular weight of 733.9 g/mol), Ethambutol hydrochloride (EMB1) (molecular weight of 277.2 g/mol), Pyrazinamide (PYR1) (molecular weight of 123.1 g/mol), Isoniazid (IZN1) (molecular weight of 137.1 g/mol) and Rifampicin (RIF1) (molecular weight of 822.9 g/mol)were purchased from DLD Scientific cc.

### 2.4 Instrumentation and experimental conditions

The LC-MS/MS equipment employed was the Sciex ExionLC AD series liquid chromatography system, connected to a Sciex 5500+ triple quadrupole mass spectrometer provided by Promolab T/A Separations (South Africa).

#### 2.4.1 Liquid chromatographic conditions

The liquid chromatography mobile phase comprised two components: acidified water (0.1 % formic acid) labelled as solvent A, and acidified methanol (0.1 % formic acid) referred to as solvent B. Chromatographic separation was achieved using a Shimadzu Shim-pack GISS C18 column (1.9 μm, 2.1 × 150 mm; Shimadzu, South Africa). The column temperature was maintained at 30 °C, and the gradient elution proceeded with the A: B solvent ratios as follows: initially at 65 % A and 35 % B for 1 min, then transitioning to 20 % A and 80 % B for 1 min, and maintaining this ratio for 3.8 min. Finally, it returned to 65 % A and 35 % B within 0.6 min and maintained this ratio for 3.6 min. The mobile phase flow rate was controlled at 0.25 mL/min, and a 2 μL sample injection volume was used (Sigma-Aldrich, 2023). A mobile phase flow rate of 0.25 mL/min is a common choice in liquid chromatography due to its balance between efficient separation and minimal column backpressure. This flow rate allows for an optimal compromise between resolving power and analysis time (Rodriguez-Aller et al., 2013 [17]). Similarly, the sample injection volume of 2 μL aligns with established chromatographic principles aimed at obtaining sharp, well-defined peaks and minimising the risk of overloading the column. Smaller injection volumes are preferred in analytical methods to prevent peak broadening and distortion, which can occur when larger volumes are injected.

#### 2.4.2 Mass spectrometry

The Sciex 5500+ mass spectrometer (MS) was equipped with a Sciex Turbo V electrospray ionisation (ESI) source, and all five compounds were detected in positive ionisation mode. Compound-dependent parameters, i.e., Declustering potential (DP), Collision energy (CE) and Collision exit potential (CXP), were optimised using the Sciex Analyst software. The optimised MS conditions and multiple reaction monitoring (MRM) acquisition transitions for each compound are presented in Table 2. The data generated through MS underwent processing with the Sciex OS data management software, and calibration graphs were subsequently generated.

**Table 2:** Sciex 5500+ mass spectrometer acquisition conditions for detecting and quantifying tuberculosis treatment drugs in wastewater.

Compound	Quadrupole 1 Mass (Da) Precursor	Quadrupole 2 Mass (Da) Product	Declustering Potential (V)	Collision exit potential (V)	Collison Energy (V)	LogP Value	Reference
Pyrazinamide	124.00	81.00	80	40	20	−0.6	[18]
Isoniazid	138.10	121.10	80	20	20	−0.7	[18]
Ethambutol	205.10	116.10	60	20	20	−0.059	[18]
Erythromycin	734.40	576.30	60	50	20	2.6	[19]
Rifampicin	824.00	792.50	40	14	20	2.95	[18]

Table 2 shows that quadrupole masses 1 and 2 represent the MRM transitions selected based on literature and further verified against the PubChem chemical database [20]. These MRM transitions were employed to refine the compound-specific parameters on the MS.

### 2.5 Quantification and method validation

To prepare stock solutions of each TB treatment drug at a concentration of 1 mg/mL, 10 mg of each respective drug was dissolved in 10 mL of methanol. These solutions were then divided into aliquots and stored at −80 °C. Calibration standards were generated by mixing equal volumes of these stock solutions and serially diluting them with methanol to achieve concentrations of 62.5 ng/mL, 31.25 ng/mL, 15.63 ng/mL, 7.81 ng/mL, 3.9 ng/mL, 1.95 ng/mL, and 0.98 ng/mL. This afforded a seven-point external calibration graph for all five compounds (Fig. 2.).

The method validation parameters were adapted from the United Nations Office on Drugs and Crime guidelines [21]. These parameters included selectivity, accuracy, linearity, limit of detection, limit of quantification and recovery of target analytes. The standards addition method was selected to evaluate matrix effects and determine the concentrations of the TB drugs in the wastewater samples. The composite standards solution was serially diluted to 50 ng/mL, 25 ng/mL, and 12.5 ng/mL. The standards for addition were then fortified to each wastewater sample in a 1:1 dilution ratio. This created a three-point standard addition calibration for each wastewater sample containing the composite first-line TB drugs. The unspiked wastewater samples were also diluted with methanol in a 1:1 ratio. The following equation computed the contribution of matrix effects (Mosekiemang et al*.,* 2019 [22]) $\%\;Matrix\;effect=\left[\;\frac{Concentration\left(spiked\right)-Blank\;\left(sample\right)}{Nominal\;concentration\;\left(spike\right)}-1\right]\times 100$.

## 3 Results

### 3.1 Chromatographic separation of selected first-line TB drugs

The first-line TB drugs selected in this study exhibited diverse polarities, as evidenced by their LogP values. For instance, Erythromycin and rifampicin had moderately polar LogP values of 2.6 and 2.95, respectively. In contrast, ethambutol, pyrazinamide, and isoniazid exhibited polar LogP values of −0.059, −0.6, and −0.7, respectively. Unlike the other antibiotics, Rifampicin had limited solubility in water, requiring all analytes to be dissolved and diluted in methanol for external calibrations.

**Fig. 1:**
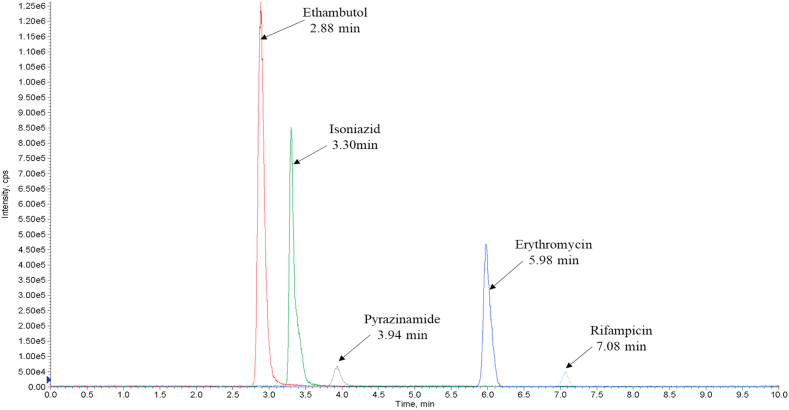
Total ion chromatogram indicating the elution times and relative intensities of five first-line TB drugs in methanol spiked with 62.5 ng/mL of each drug.

Moreover, potential interferences were resolved, resulting in analyte peaks evenly distributed throughout the 10-min chromatographic period. Ethambutol was eluted at 2.88 min, followed by isoniazid at 3.30 min. Subsequently, pyrazinamide, erythromycin, and rifampicin were eluted at 3.94, 5.98, and 7.08 min, respectively (Fig. 1). This sequence corresponds to the analytes' relative polarities, with more polar molecules (ethambutol, isoniazid, and pyrazinamide) eluting first, followed by the moderately polar erythromycin and rifampicin. This alignment aligns with the expectations of reversed-phase chromatography, where moderately polar to non-polar analytes are retained longer on the column. It is important to note that signal intensities for pyrazinamide and rifampicin were lower than other target analytes, but successful calibrations were achieved for all analytes, ensuring reliable results (Fig. 1).

**Fig. 2:**
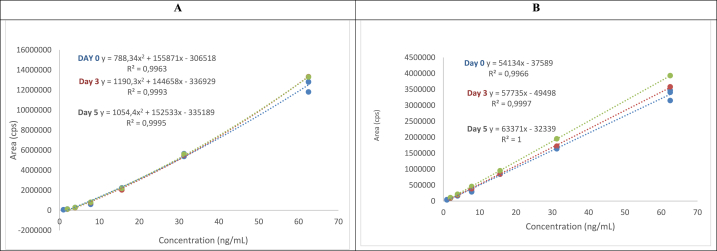
Stability of ethambutol (A) and isoniazid (B) after 1:1 water to methanol dilution stored at four °C.

### 3.2 Quantification and matrix effects of TB drugs in wastewater samples

The correlation coefficient (R^2^) for all TB drugs in the wastewater samples, obtained by spiking them with known concentrations of these antibiotics and subjecting them to the entire analytical procedure, consistently yielded a high value of 0.99, except for rifampicin in treated wastewater samples from WWTP A (Table 3). The measured R^2^ indicates that increasing the concentration of these TB antibiotic standards spiked into the wastewater was associated with increased concentration of these drugs detected in the wastewater. This suggests that the wastewater matrix did not significantly impact the concentration of these drugs in the wastewater samples. Therefore, this finding indicates that the antibiotic concentration detected reflects the concentration entering the wastewater environment and is not masked by matrix effects.

**Table 3:** Results of linear standards addition calibration, LOD, LOQ, and matrix effects for analytes in various samples.

Sample	Erythromycin				Ethambutol				Pyrazinamide				Isoniazid				Rifampicin			
	R^2^	LOD	LOQ	%ME	R^2^	LOD	LOQ	%ME	R^2^	LOD	LOQ	%ME	R^2^	LOD	LOQ	%ME	R^2^	LOD	LOQ	%ME
WWTP An untreated	0.99	0.66	2.19	−18	0.99	1.84	6.12	56	0.99	3.23	10.76	−6	0.99	3.07	10.25	−55	0.99	0.2	0.5	−15
WWTP A Treated	0.99	0.88	2.93	−19	0.99	0.64	2.12	36	0.99	3.96	13.19	−11	0.99	1.33	4.43	−1	nd	nd	nd	−65
WWTP D Untreated	0.99	2.85	9.50	−19	0.99	3.01	10.04	75	0.99	2.95	9.83	−7	0.99	2.92	9.72	−142	0.99	2.37	7.91	−12
WWTP D Treated	0.99	0.93	3.09	−19	0.99	3.94	13.15	70	0.99	1.47	4.90	−11	0.99	1.07	3.56	1	0.99	2.38	7.94	−38
WWTP B Untreated	0.99	0.95	3.16	−18	0.99	2.78	9.26	24	0.99	1.99	6.62	−6.4	0.99	2.60	8.68	−3	0.99	1.63	5.44	−28
WWTP B Treated	0.99	2.64	8.81	−38	0.99	3.31	11.05	65	0.99	2.10	6.99	−10	0.99	2.39	7.96	9	0.99	2.64	8.81	−38
WWTP E Untreated	0.99	2.75	9.16	−21	0.99	1.03	3.43	33	0.99	3.40	11.32	−12	0.99	1.59	5.28	18	0.99	1.27	4.23	−26
WWTP E Treated	0.99	1.37	4.56	−16	0.99	0.26	0.88	72	0.99	2.73	9.01	−8	0.99	1.02	3.41	11	0.99	4.05	13.5	−51
WWTP F Untreated	0.99	4.01	13.4	−17	0.99	2.01	6.71	39	0.99	3.32	11.05	−8	0.99	4.20	14.01	−81	0.99	2.61	8.69	−21
WWTP F Treated	0.99	3.29	11	−17	0.99	1.49	4.98	13	0.99	3.53	11.77	−16	0.99	1.89	6.29	6	0.99	2.81	9.36	−36
WWTP C Treated	nd	nd	Nd	−21	0.99	2.26	7.54	53	0.99	3.73	12.42	−6	0.99	3.90	13.00	4	0.99	2.08	6.95	−42

NB*. [A]: denotes the concentration of the analyte calculated via the standards addition method.

R^2^: describes the correlation coefficient for linear, standard addition calibrations.

LOD: describes the detection limit (ng/mL) for each analyte computed using the standards addition method.

LOQ limit of quantification (ng/mL) for each analyte computed from the limit of quantification (ng/mL) for each analyte calculated using the standards addition method.

%ME average percentage contribution of the matrix effect across the three-point standards addition. Negative values denote analyte suppression, whereas positive values indicate analyte enhancement.

nd: denotes values that could not be computed.

The limit of detection (LOD) for the various antibiotics, which indicates the sensitivity of the analytical method, varied significantly, showing a variation in method sensitivity. For instance, the LOD for erythromycin ranged from 0.66 ng/mL to 4.01 ng/mL, with an average of 2.03 (±1.21) ng/mL in the untreated wastewater samples. Similar variations were observed for the other antibiotics, irrespective of treated or untreated wastewater (Table 3). For example, in treated wastewater, LOD for ethambutol ranged from 0.26 ng/mL to 3.94 ng/mL, averaging 2.05 (±1.15) ng/mL. Pyrazinamide LODs ranged from 1.47 ng/mL to 3.96 ng/mL, averaging 2.95 (±0.79) ng/mL. It is worth noting that the lowest LOD of 0.2 ng/mL was observed for rifampicin in untreated wastewater (Table 3),

The Limit of Quantification (LOQ), a crucial parameter in analytical method development, which represents the lowest concentration or amount of an analyte in a sample that can be reliably quantified with acceptable accuracy and precision, also varied among the antibiotics measured in wastewater. For instance, the LOQ for erythromycin ranged from 1.4 ng/mL to 11 ng/mL, with an average of 5.58 (±3.61) ng/mL. Ethambutol LOQ ranged from 0.88 ng/mL to 13.15 ng/mL with an average LOQ of 6.84 (±3.84) ng/mL. It is also worth mentioning that the lowest LOQ was 0.5 ng/mL for RIF, corresponding to the low LOD discussed above (Table 3).

Assessment of the matrix contribution or matrix effect (%ME) also showed that these antibiotics are affected differently by other components within the wastewater. For instance, a matrix contribution (%ME) ranging from −38 to −16, with an average of −20.27 (±6.08) for erythromycin, was observed. This indicates that the wastewater matrix suppressed the concentration of the erythromycin detected. Similar effects were observed for pyrazinamide, isoniazid and rifampicin. However, the observed matrix contribution (%ME) for ethambutol ranged from 13 to 75, averaging 48.72 (±21.01). A similar effect was observed for isoniazid in untreated wastewater from WWTP E (Table 3). This indicates that the wastewater matrix increased the concentration of these antibiotics. A Kruskal-Wallis Test showed statistically significant differences in LOD, LOQ, and matrix contribution between the analytes and the sampling sites (p < 0.05).

### 3.3 Stability of TB drugs in wastewater during storage at 4 °C

**Fig. 3:**
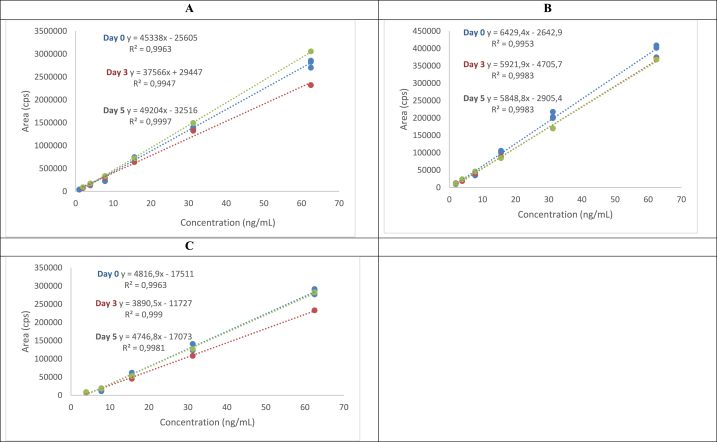
Stability of erythromycin (A), pyrazinamide (B) and rifampicin (C) after 1:1 water to methanol dilution stored at four °C.

The impact of storage on ethambutol over five days was also evaluated, considering the short-term nature of storage. The obtained R^2^ values at different time points, including day 0, day 3, and day 5, were 0.9963, 0.9993, and 0.9995, respectively. These R^2^ values measured the correlation between the concentration of the TB drugs detected and the known concentrations spiked into the wastewater samples. This was done to determine any loss of TB drug concentrations during wastewater storage. This examination also extended to other TB drugs, with a consistent pattern when assessing the influence of storage at 4 °C. Fig. 2, Fig. 3 present this trend effectively. A notable example is pyrazinamide, for which the R^2^ values stood at 0.9953, 0.9983, and 0.9983 for day 0, day 3, and day 5, respectively (Fig. 2B). It is essential to highlight that the loss of TB drugs throughout this process remained below 1 log, attesting to the fact that the detection efficiency remained largely unaltered by the storage protocols.

### 3.4 Concentration of TB drugs in untreated and treated wastewater

Applying the protocol in treated and untreated wastewater from the various countries yielded varying results (Table 4). For instance, erythromycin was detected in three untreated samples, but concentrations were below the LOQ. Ethambutol was consistently found in all samples except WWTP C, with concentrations ranging from 2.64 ng/mL to 10.11 ng/mL. It is worth mentioning that pyrazinamide and isoniazid had low detection in untreated wastewater (Table 4). The detection of these antibiotics in the treated wastewater samples indicated potential surface water contamination. For instance, treated wastewater from WWTP F contained 2.64 ng/mL of ethambutol, WWTP B had pyrazinamide at 9.44 ng/mL, and rifampicin at 4.27 ng/mL. It is also imperative to note that some antibiotics remained undetected in the treated wastewater samples, preventing removal efficiency calculation.

**Table 4:** Summary of results of six individual wastewater treatment works (WWTP) indicating the concentration (ng/mL) of TB treatment drugs in the untreated (inf) and treated (eff) wastewater.

WWTP	Erythromycin (ng/mL)		Ethambutol (ng/mL)		Pyrazinamide (ng/mL)		Isoniazid (ng/mL)		Rifampicin (ng/mL)	
	*Inf*	*Eff*	*Inf*	*Eff*	*Inf*	*Eff*	*Inf*	*Eff*	*Inf*	*Eff*
WWTP A	1.21^a^	Nd	8.25	8.28	Nd	nd	nd	Nd	2.60	nd
WWTP D	Nd	2.68^a^	10.11	10.2^a^	Nd	3.31^a^	nd	Nd	4.18^a^	nd
WWTP B	3.09^a^	1.63^a^	7.54^a^	9.44^a^	2.21^a^	nd	nd	Nd	3.11^a^	4.27^a^
WWTP E	3.50^a^	2.52^a^	7.43	8.68^a^	Nd	nd	54.61	1.27^a^	2.53^a^	nd
WWTP F	Nd	Nd	8.09	2.64^a^	Nd	nd	nd	Nd	nd	nd
WWTP C	Nd	Nd	Nd	8.42	Nd	nd	nd	Nd	nd	4.48^a^

NB^a^. ***Inf, Eff*:** denotes influent/untreated wastewater and effluent/treated wastewater, respectively.

**nd**. Not detected.

a denotes concentrations more significant than the LOD values but less than the LOQ values.

## 4 Discussion

The utilisation of Wastewater-Based Epidemiology (WBE) as a tool for monitoring antibiotic consumption, particularly in tuberculosis (TB) treatment, presents an innovative approach to public health surveillance. The methodology, while promising, is contingent upon the accurate and precise identification and quantification of antibiotic residues in wastewater, a task riddled with complexity due to several critical factors. This discussion aims to delineate these complexities, drawing from the current study's findings and situating them within the broader scientific discourse.

The study at hand delves into the intricate interplay between the concentration of TB antibiotics in wastewater and their chemical properties, such as polarity, which are seminal in determining their detectability and quantifiability. The exploration of the polarities and chromatographic behaviours of critical TB antibiotics—erythromycin, rifampicin, ethambutol, pyrazinamide, and isoniazid—underscores the nuanced nature of this endeavour. Echoing the sentiments of DeGorter et al. [23] and subsequent corroborations by Sims and Kasprzyk-Hordern [24] and Prahl et al. [25], the study reaffirms the critical role of polarity in the environmental detection of these compounds. The dissolvability challenges of rifampicin in aqueous solutions further accentuate the need for methodological rigour in solvent selection, emphasising methanol's suitability for rifampicin based on its compatibility with chromatographic analysis and findings related to solubility studies (Zheng et al., 2020 [26]).

Signal intensity variations, particularly for rifampicin and pyrazinamide, present another layer of analytical complexity, hinting at the multifarious factors—ranging from environmental conditions to matrix effects—that can influence analytical outcomes. This study's in-depth examination sheds light on the multifaceted nature of detecting antibiotics in environmental samples, resonating with findings from De Nicolò et al. [27], Fei et al. [28], and others who have traversed similar investigational terrains ([29,30]. Like other studies ([27]; [28]; [25,[31], [32], [33]]), this study provides insights into the complexities affecting signal intensity in environmental samples.

An intriguing aspect of this research is the observed discrepancy in the correlation of rifampicin concentrations in treated wastewater from a specific treatment facility. This points towards the potential quirks of wastewater treatment processes and their impact on drug detectability. This divergence calls for a tailored approach to method optimisation and underscores the importance of context-specific investigations into the behaviour of TB drugs in varied wastewater treatment paradigms.

Moreover, the variations in detection limits (LOD and LOQ) across different samples spotlight the significance of accounting for the unique characteristics of each sample matrix. This consideration is paramount in navigating the analytical hurdles posed by the presence of organic matter, inorganic salts, and particulate matter within wastewater, which can introduce interference, alter drug ionisation and chromatographic behaviour, and affect bioavailability for detection ([34]; [35]; [36,37]).

The matrix effects measured in this study, denoted by the matrix contribution (%ME), elucidate the substantial impact of the sample matrix on TB drug analysis. The observed suppression effects and the variability in %ME values across sampling sites and drugs illuminate the heterogeneity of wastewater matrices, further complicating the analytical landscape. The %ME values reveal the sample matrix's influence on TB drug analysis. Negative %ME values indicate matrix suppression effects, which are consistent with prior findings for pharmaceuticals in environmental samples [18,[38], [39], [40], [41]].

The exploration of ethambutol's storage and detection efficiency, among other TB drugs, reinforces the methodological soundness of the methanol extraction and storage protocol employed. Yet, it also opens avenues for future research to optimise analytical methodologies for diverse sample matrices to ensure the accurate quantification of TB drugs in various environmental contexts. Additionally, understanding how different sample matrices affect drug stability and detection efficiency during storage and extraction is essential for optimising analytical methodologies for accurate TB drug quantification in various sample types [19,31]. These insights support the development of guidelines and protocols for TB drug analysis, both in clinical and environmental contexts, enhancing the reliability of drug quantification methods and ensuring the integrity of data in diverse applications ([[42], [43], [44], [45]]; [46]).

The findings reported in this study indicate that wastewater analysis could act as a snapshot of TB drug consumption within connected populations. This is by previous studies (Verlicchi et al., 2015 [38,39]; [[47], [48]] [49]; [12,13,50,51]). However, it is worth noting that this study also highlights that WWTPs could accumulate these drugs in the wastewater systems, potentially resulting in increased risks of exposure by both microorganisms and contributing to antimicrobial resistance development.

Finally, the differential occurrences and removal efficiencies of TB drugs in wastewater samples spotlight the environmental footprint of these antibiotics and raise pertinent questions regarding their potential to contribute to antimicrobial resistance. The variation in drug concentrations and the efficacy of wastewater treatment processes in mitigating drug accumulation necessitate careful interpretation of WBE data and the development of refined strategies for drug removal and WBE application. This study contributes to the burgeoning field of WBE and charts a course for future research dedicated to enhancing the precision, accuracy, and applicability of WBE in monitoring TB drug consumption and, by extension, combating antibiotic resistance. Further investigative efforts are warranted to unravel the intricacies of drug occurrence, fate, and accumulation in wastewater, ultimately paving the way for optimising removal strategies and refining WBE as a cornerstone of public health surveillance.

## 5 Conclusion

This study optimised an analytical method for detecting TB-related drugs in wastewater. The optimised method considered the polarities of the standard first-line TB drugs, which is critical for detection and quantification using the LCMS/MS technology. Furthermore, the effect of the sample matrix on the detection and quantification of these drugs was determined and shown not to significantly impact the concentration of the drugs detected based on the R^2^ calculated using spiked wastewater. However, the matrix contribution (%ME) showed that the wastewater matrix primarily suppressed the concentration of these drugs. The application of this method across four African countries showed that TB drug concentration in wastewater from these countries varied, which could be attributed to the difference in drug consumption in these countries or other factors such as the sample matrix. Factors such as the fate of these drugs in the wastewater system and removal during wastewater treatment could have also accounted for the differences observed.

In conclusion, this study provides valuable insights into factors that need to be accounted for when applying WBE for antibiotic consumption monitoring. The findings also contribute significantly to the existing literature on the occurrence and removal of TB drugs in wastewater while raising important questions for future research. Further investigations are necessary to gain a deeper understanding of the factors influencing drug occurrence, optimise treatment processes, and refine WBE for monitoring TB drug consumption in low—and middle-income countries.

## Limitations

Despite the valuable insights provided by this study, it is essential to acknowledge certain limitations that may affect the interpretation and generalizability of the findings.

1. **Geographic limitation:** The study's conclusions are based on wastewater samples from four African urban areas, which may not represent other locations with differing healthcare practices and infrastructure.
2. **Sample Representativeness:** Because of shifting consumption patterns, the restricted sampling frequency and duration may fail to capture the variability in TB drug presence correctly.
3. **Method Specificity:** Using a single analytical method for tuberculosis medications may limit data comparability with other studies and fail to account for environmental variability or matrix effects.
4. **Treatment Process Analysis:** The study gives limited insight into the efficacy of wastewater treatment methods for drug removal, which should be explored further in future research.
5. **Chemical Stability:** The study did not extensively explore the stability of TB drugs under diverse environmental conditions or the impact of matrix effects.
6. **Source Tracing:** There was no in-depth investigation into pinpointing the exact origins of TB drugs in wastewater, such as differentiating between hospital and domestic sources

## Recommendations and future research

Based on the findings and limitations of this study, the following recommendations are proposed to advance the field of TB drug analysis in wastewater and further our understanding of their occurrence, behaviour, and environmental impact.

1. **Broader Geographical Coverage:** Include a more diverse set of locations to enhance the generalizability of results across different socio-economic and healthcare settings.
2. **Enhanced Sampling Strategy:** Employ longer and more frequent sampling to better understand TB drug presence's temporal and seasonal dynamics.
3. **Diverse Analytical Approaches:** Use and compare multiple analytical methods to ensure the findings' broader applicability and account for methodological sensitivities.
4. **Broader Drug Spectrum:** Expand the investigated pharmaceutical range to include various TB treatment options.
5. **In-Depth Process Study:** Conduct detailed research into the efficacy of specific wastewater treatment stages in eliminating TB drugs.

## Data availability

The data pertinent to this study have not been deposited in a publicly accessible repository. Nevertheless, all relevant data are comprehensively outlined within the article, accompanied by supplementary materials or appropriately referenced within the manuscript. Additional data can be made accessible upon request.

## CRediT authorship contribution statement

**Hlengiwe N. Mtetwa:** Writing – review & editing, Writing – original draft, Investigation, Formal analysis, Data curation, Conceptualization. **Isaac D. Amoah:** Writing – review & editing, Supervision, Investigation, Formal analysis, Data curation, Conceptualization. **Sheena Kumari:** Writing – review & editing, Supervision, Funding acquisition, Conceptualization. **Faizal Bux:** Writing – review & editing, Resources, Funding acquisition, Conceptualization. **Poovendhree Reddy:** Writing – review & editing, Supervision, Project administration, Investigation, Funding acquisition, Data curation, Conceptualization.

## Declaration of competing interest

The authors declare that they have no known competing financial interests or personal relationships that could have appeared to influence the work reported in this paper.
